# Healing of surgical site after total hip and knee replacements show similar telethermographic patterns

**DOI:** 10.1007/s10195-011-0135-1

**Published:** 2011-05-05

**Authors:** Carlo Luca Romanò, Delia Romanò, Francesca Dell’Oro, Nicola Logoluso, Lorenzo Drago

**Affiliations:** 1 Dipartimento di Chirurgia Ricostruttiva e delle Infezioni Osteo-articolari, Istituto Ortopedico I.R.C.C.S. Galeazzi, Via Riccardo Galeazzi, 4, 20161 Milan, Italy; 2 Laboratorio analisi e microbiologia, Istituto Ortopedico I.R.C.C.S. Galeazzi, Via Riccardo Galeazzi, 4, 20161 Milan, Italy

**Keywords:** Thermography, Hip, Knee, Wound, Infection

## Abstract

**Background:**

Isolated reports indicate the efficacy of infrared thermography for monitoring wound healing and septic complications, but no long-term analysis has ever been performed on this, and there are no data on the telethermographic patterns of surgical site healing after uncomplicated total hip prosthesis and after knee prosthesis.

**Materials and methods:**

In this prospective, observational, nonrandomized cohort study, two groups with forty consecutive patients each, who were operated on respectively for total hip and for total knee replacements, underwent telethermographic examination of the operated and contralateral joints prior to and at fixed intervals for up to 1 year after uncomplicated surgery. A digital, portable telethermocamera and dedicated software were used for data acquisition and processing.

**Results:**

No thermographic difference was observed preoperatively between the affected side and the contralateral side in both groups. After the intervention, a steep increase in the temperature of the operated joint was recorded after total hip replacement and after knee replacement, with a peak mean differential temperature measured three days postoperatively between the operated and unoperated joint of 3.1 ± 0.8°C after total hip replacement, and 3.4 ± 0.7°C after total knee replacement. Thereafter, the mean differential temperature declined slowly to 0.7 ± 1.1°C and to 0.5 ± 1.3°C at 60 days, and to 0.0 ± 1.0°C and −0.1 ± 1.1°C 90 days post-operatively, respectively. No further changes were observed for up to 1 year after surgery. Results were similar when comparing the average telethermographic values of an elliptical area where the main axis corresponded to the surgical wound.

**Conclusions:**

The surgical sites after uncomplicated total hip or total knee replacement show similar telethermographic patterns for up to 1 year from surgery, and can easily be monitored using a portable, digital, telethermocamera.

## Introduction

Infrared thermography (IRT) has been shown to detect temperature changes associated with many different diseases [1–6] and to be useful for the postoperative monitoring of surgical site healing in various clinical settings [7–12]. To our knowledge, only one paper—published nearly two decades ago—has described the early thermographic changes at the surgical site following orthopedic surgery [13], while only a few others have reported on the use of this technology for the diagnosis of bone and joint infections [14–16].

This paucity of data and the methodological limitations of such studies (different recording technologies, short follow-up, limited series of patients, lack of controls, etc.), together with the technological limits of previously available technologies (weight, portability, need for skin contact, complex data processing, costs, etc.), have prevented the wider introduction of IRT into clinical practice, despite evidence of a reproducible thermographic pattern of wound healing during the very early stages following surgery and reports of a persistent temperature rise in the presence of septic complications at the surgical site.

Infrared digital telethermography (IRDT) can now be performed through newly available digital telethermocameras [17], which offer portability, ease of use (even for nonspecialized personnel), and precise and real-time measurements at relatively low cost.

Total hip and knee replacements are among the most widely performed and successful interventions in orthopedics, with more than one million new prostheses implanted each year in Western countries; however, wound healing complications and surgical site infections remain difficult to diagnose [18–20] and challenging and expensive to treat [21–23], while the clinical assessment of wound healing is unreliable [24].

The purpose of this prospective study was to assess and compare the physiological telethermographic pattern of surgical site healing of uncomplicated total hip replacement (THR) with the corresponding pattern for total knee replacement (TKR) for an extended period of time after surgery, in order to set a reference value for further, future, analysis and to elucidate if the healing pattern depends on the location of the healing site on the body, or if it is largely the same whatever the healing site location.

The equipment used consisted of a portable and lightweight digital infrared thermocamera, which permitted data acquisition at the patient’s bedside or on an outpatient basis after patient discharge from the hospital, as well as dedicated software for data processing and analysis.

## Materials and methods

During the year 2008, two groups of forty patients that underwent THR or TKR, respectively, were included in this prospective, observational, nonrandomized cohort study. The study was approved by the Institutional Review Board and conformed to the Declaration of Helsinki as amended in 2008.

Preoperative diagnoses of the hip group were: primary coxarthrosis in 22, hip dysplasia in 13, and femoral head necrosis in 5 patients; in the knee group: primary osteoarthritis (32 patients), post-traumatic osteoarthritis (4), and osteonecrosis (4). Patients with previous surgery at the same joint, rheumatological disorders, peripheral vasculopathies, or a history of previous joint infections were excluded.

The surgical procedure was similar in all patients. In the hip group, an uncemented total hip prosthesis (Recta, AdlerOrtho S.p.A., Milan, Italy) was implanted through a direct lateral approach, with the patient laying supine. In the knee group, a cemented total knee arthroplasty (PFC, Johnson & Johnson-DePuy Inc., Warsaw, IN, USA) was implanted, including patella resurfacing, through a midline skin incision and a medial parapatellar approach. Patients of both groups received enoxaparin 0.4 ml/day for 35 days after surgery to prevent thromboembolic complications. Short-term antibiotic prophylaxis was performed in all cases with cefotaxime. No drains were used after surgery in either group.

Partial weight bearing with two crutches was allowed for 6 weeks, followed by one-crutch weight bearing for 4–6 weeks. Full weight bearing was permitted at 10–12 weeks after surgery.

The clinical evaluation included the Harris hip score and the Knee Society score evaluated prior to and 6 and 12 months after surgery, as well as the recording at each follow-up of clinical signs of infection (redness, swelling, pain, joint stiffness, draining fistulas) for up to 2 years after surgery.

Serum C-reactive protein (C-RP) and erythrocyte sedimentation rate (ESR) were tested prior to the intervention and twice a week after surgery until the 20th postoperative day, and then monthly for 3 months.

All patients also underwent a standard X-ray examination of the operated limb prior to, immediately after, and at 3 and 12 months following surgery. They also all gave their informed consent to participate in the study, which was partially funded by the Italian Ministry of Health (research project no. 4021/08).

### Telethermographic data acquisition and processing

The IR images were acquired with the NEC-AVIO ThermoShot F30S digital telethermocamera. The specifications of this camera were as follows: measurement range: −20 to 100°C; temperature resolution: 0.1°C (at 30°C), better than 0.1°C with averaging; wavelength: 8–13 μm; spatial resolution: 3.1 mrad; measurement distance: 10 cm to infinity; dimensions: 100 × 65 × 45 mm; weight: 350 g, including rechargeable batteries.

**Fig. 1:**
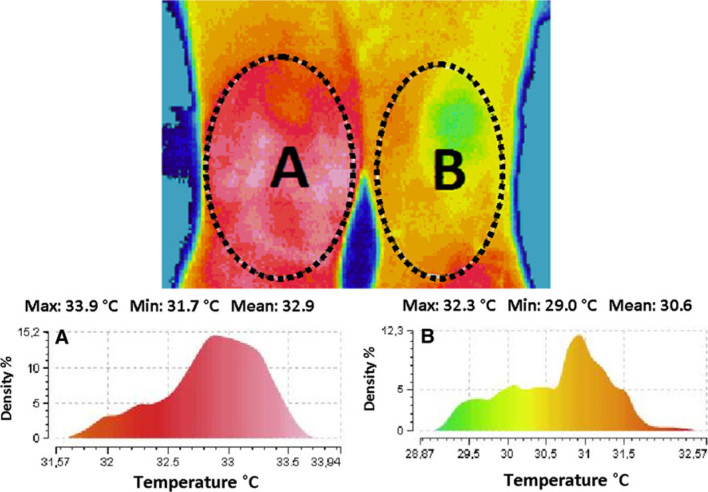
Postoperative telethermographic image 8 days after TKR (*right knee* is the operated joint). The differential temperature measured at the hottest spot (HS) is 1.6°C, while that for the surgical site area (SSA), as indicated by the *dotted line*, is 2.3°C

Data acquisition was performed the day before or on the day of surgery and then after surgery at days 1, 3, 8, 13, 15, 30, 45, 60, 90, 180, and 365. The telethermographic images used for further analysis in this study included only the surgical site area and the corresponding area on the contralateral limb. An elliptical area was drawn with its major axis on the surgical wound, extended approximately 10 cm proximally and distally, and given a minor axis that was approximately 12 cm in length and crossed the major axis at the midline (Fig. 1).

Thermographic images were acquired with the patient laying supine. One shot was taken from each location. For the hip, the first shot was taken of the surgical site area, and then one was taken of the contralateral hip, while for the knee it was possible to acquire both joints with the same shot. Data for the operated limb were compared to data for the contralateral side. No effort was made to keep the ambient temperature or humidity at a constant level, as all of those parameters were automatically recorded by the digital camera and considered to affect both limbs equally at the instant when the temperature was recorded. However, care was taken to leave the joints uncovered for at least three minutes before each recording, and to avoid liquid dressings on the joint and direct illumination of the joint from spotlights during thermographic data acquisition.

The thermographic images were evaluated with the dedicated software IRTCronista.

Primary outcomes included the mean absolute temperature and the mean differential temperature (MDT, affected minus unaffected values) for all of the patients, measured at the:

- Surgical site area (SSA): the elliptical area described above
- Hottest spot (HS): the location corresponding to the highest temperature inside the SSA

Secondary outcomes were a comparison of time-dependent thermographic changes with serum C-reactive protein and erythrocyte sedimentation rate.

Statistical analysis was performed with the unpaired Student’s *t* test, with the level of significance set at *P* < 0.05.

## Results

Demographic data for the hip and the knee groups were as follows: males versus females: 27:13 and 28:12; mean age at time of operation: 65.3 years (range, 54–76 years) and 63.5 years (range, 53–78 years). In the hip group, there were 4 smokers, while 3 patients were affected by diabetes mellitus and 1 had renal insufficiency; in the knee group, 6 patients were smokers, while 2 more had diabetes mellitus. No other major comorbidities were found.

No thermographic differences were observed preoperatively between the affected joint and the contralateral joint, whatever the preoperative diagnosis and the affected joint. The preoperatively recorded mean absolute hottest spot (HS) temperatures were 33.6 ± 0.9°C and 33.6 ± 1.0°C (affected and contralateral hips), and 33.8 ± 0.7°C and 33.7 ± 0.5°C (affected and contralateral knees). The preoperatively recorded mean surgical site area (SSA) temperatures were, respectively, 32.0 ± 1.0°C and 31.9 ± 1.1°C at the hip and 32.2 ± 1.0°C and 32.0 ± 1.1°C at the knee.

**Fig. 2:**
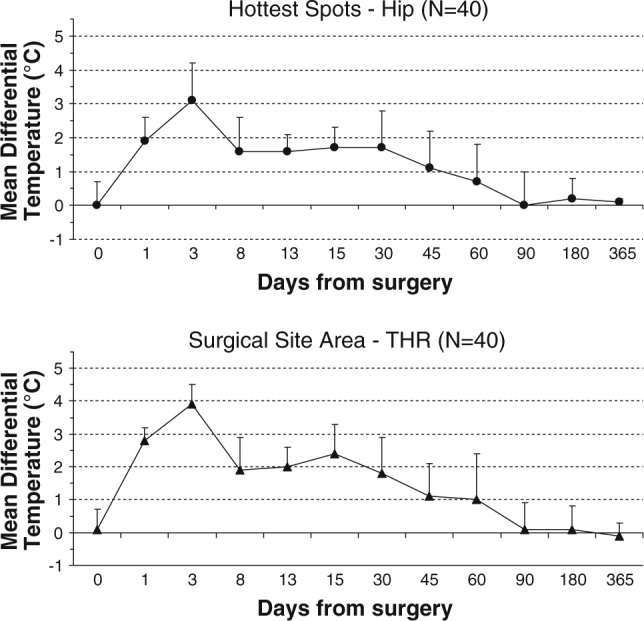
Telethermographic pattern of surgical site healing after THR. Mean differential temperatures (+ standard deviations) of the HS and the SSA. The values at zero on the *x*-axes indicate preoperative data

**Fig. 3:**
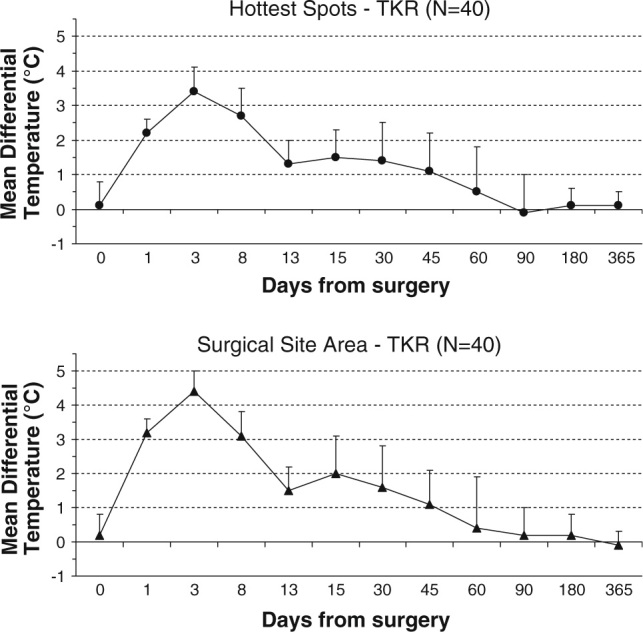
Telethermographic pattern of surgical site healing after TKR. Mean differential temperatures (+ standard deviations) of the HS and the SSA. The values at zero on the *x*-axes indicate preoperative data

After the intervention, a steep increase in the temperature of the operated joint was observed in every patient. The peak in the mean differential temperature (MDT, affected minus unaffected values) occurred on the third postoperative day, and the MDT slowly returned to baseline levels 90 days after surgery in both the THR and TKR groups (Figs. 2, 3). No further changes were seen in the MDT at longer follow-up.

The MDT measured on the third postoperative day was slightly lower at the hip than at the knee. In fact, the MDTs of the hip HS and SSA, were, respectively, 3.1 ± 1.1°C and 3.9 ± 0.6°C, while those for the knee were 3.4 ± 0.7°C and 4.4 ± 0.6°C. No statistical difference was observed (*P* > 0.05) between the mean telethermographic values for the hips and knees seen on any particular postoperative day.

The temperature of each hottest spot is, by definition, higher then the average temperature calculated for the corresponding surgical site area. The mean differences between all of the recorded hottest spot temperatures and all of the mean surgical site area temperatures were 1.5 ± 0.6°C for the operated hip and 1.4 ± 0.7°C for the operated knee. In the nonoperated limb, this difference was the same for the hip and the knee (1.7° ± 0.6°C).

**Fig. 4:**
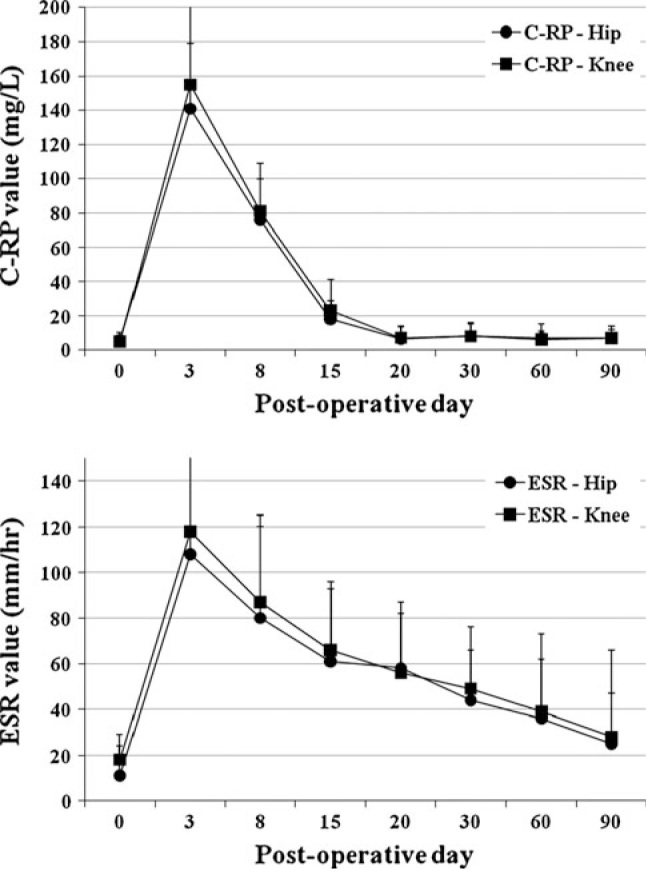
C-reactive protein (C-RP) and erythrocyte sedimentation rate (ESR) time courses after THR and TKR (mean values + standard deviation)

The mean preoperative serum C-reactive protein levels were 5.6 ± 4.8 mg/l and 4.8 ± 3.8 mg/l, respectively, in the hip and knee groups; peaks of 141 ± 38 mg/l and 155 ± 48 mg/l, respectively, were observed on the third postoperative day, while the levels returned to baseline (6.6 ± 7.0 mg/l and 7.1 ± 6.8 mg/l, respectively) 20 days after surgery. Erythrocyte sedimentation rate levels were, respectively, 11 ± 18 mm/h and 18 ± 6 mm/h preoperatively, and 25 ± 22 mm/h 28 ± 38 mm/h three months after surgery (Fig. 4).

The Harris hip score improved from 38.4 ± 14.5 to 92.3 ± 14.4 after THR, while the Knee Society score was 68 ± 19 pre-operatively and 91 ± 12 one year after TKR. No patient showed any clinical or laboratory signs of infection right up to the most recent follow-up. None of the patients required any further surgery, and X-ray examinations did not indicate any sign of early loosening of the prosthesis.

## Discussion

While various studies have shown the ability of infrared thermography to monitor wound healing after surgery and to screen for early postsurgical infections, this technology is still to be widely introduced to clinical practice. In our opinion, this has mainly been due to a paucity of data, the methodological limitations of previous studies, and to the technological limits of previously available thermocameras.

The advent of more advanced infrared digital telethermocameras, with their dedicated software, offer a new opportunity to make good use of this noninvasive, low-cost, safe and repeatable imaging technology in the clinical setting. To achieve this aim, the setting of reference values is mandatory for any further step, especially those concerning the use of this technology to screen out postsurgical infections, for which early detection and treatment may yield better prognoses and allow less invasive treatments [21].

Our study follows on from the only other available reportc (to our knowledge), which was published two decades ago [13], on thermographic wound monitoring after orthopedic surgery.

The present analysis confirms and extends previous observations [11–16] indicating there is a reproducible pattern to the time course of the temperature at the surgical site, with the peak temperature occurring the first few days after surgery. In our study, however, the return to the baseline temperature took considerably longer than was previously reported (a few days); in fact, the telethermographic pattern looks similar to the kinetics of C-RP during the first week [25], but baseline levels were reached over a period of time that was closer to that of the ESR.

Our study shows that different surgical interventions and body locations show similar telethermographic patterns in terms of both the mean calculated differential temperature and the time course. Knowledge of the physiological thermographic pattern may serve as a reference for further studies that focus on telethermography as a tool for rapidly screening for septic complications after surgery.

This study also points out that there is a mean difference of approximately 1.5°C between the temperature of the hottest spot and the average value of the corresponding surgical site area in both the hip and the knee, and in the nonoperated leg and the operated leg. However, if we consider the mean differential temperature between the operated and nonoperated limb, the telethermographic patterns of the hottest spots and the surgical site areas look very similar. This observation, if confirmed, suggests that routine wound monitoring could be performed by simply recording the hottest spot in the clinical setting.

To our knowledge, this is the largest study and the study with the longest follow-up on the use of telethermography to monitor surgical site healing in orthopedic surgery. However, there are many shortcomings to this study:

- The main purpose of this study was to set reference values for the physiological telethermographic patterns of surgical site healing after THR and TKR, and to compare them. The series of patients excluded those with rheumatological diseases, peripheral vasculopathies, previous surgeries and infections. We do not know if and how these variables influence the telethermographic data preoperatively or after surgery.
- We do not know if and to what extent the reported results can be appiled to other surgical procedures.
- Although various reports in the literature [12, 13, 16] point out that a lack of a drop in temperature soon after surgery or a second peak in the local temperature may indicate local septic complications, it was not the aim of the present study to investigate such phenomena, and they should be confirmed in separate research. The same notion also applies to possible differential diagnoses (deep vein thrombosis, aseptic loosening, etc.) that may lead to thermal changes at the surgical site.

Even with its limitations, this study shows that the advantages of telethermovision for observing temperatures during primary healing at a surgical site are manifold. The method is reproducible, painless, safe, does not require contact (thereby decreasing the risk of contamination from outside), gives an absolutely accurate image of the temperature over large or small areas, and the analysis of the thermogram is a relatively simple task. Follow-up examinations are easily performed during outpatient visits and are relatively quick to perform.

Defining the healing process as changes in the thermal distribution in terms of intensity, extensiveness and time permits the possibility of accurately and indirectly observing biochemical and chemicophysical changes in the wound and its surroundings, and correlates well with serological markers of inflammation.

Today’s digital telethermocameras are lightweight, portable, easy to use (even for nonspecialized personnel), relatively inexpensive, and appear to be good candidates for use as large-scale surgical site screening tools in the hospital as well as in the physician’s office.
